# Hypoxia Depresses Synaptic Transmission in the Primary Motor Cortex of the Infant Rat—Role of Adenosine A_1_ Receptors and Nitric Oxide

**DOI:** 10.3390/biomedicines10112875

**Published:** 2022-11-10

**Authors:** Isabella Zironi, Giorgio Aicardi

**Affiliations:** 1 Department of Physics and Astronomy, University of Bologna, 40127 Bologna, Italy; 2 Department for Life Quality Studies, University of Bologna, 40127 Bologna, Italy

**Keywords:** hypoxia, postnatal asphyxia, motor cortex, synaptic transmission, adenosine, adenosine A_1_ receptor, nitric oxide, cGMP, infant rat, neuroprotection

## Abstract

The acute and long-term consequences of perinatal asphyxia have been extensively investigated, but only a few studies have focused on postnatal asphyxia. In particular, electrophysiological changes induced in the motor cortex by postnatal asphyxia have not been examined so far, despite the critical involvement of this cortical area in epilepsy. In this study, we exposed primary motor cortex slices obtained from infant rats in an age window (16–18 day-old) characterized by high incidence of hypoxia-induced seizures associated with epileptiform motor behavior to 10 min of hypoxia. Extracellular field potentials evoked by horizontal pathway stimulation were recorded in layers II/III of the primary motor cortex before, during, and after the hypoxic event. The results show that hypoxia reversibly depressed glutamatergic synaptic transmission and neuronal excitability. Data obtained in the presence of specific blockers suggest that synaptic depression was mediated by adenosine acting on pre-synaptic A_1_ receptors to decrease glutamate release, and by a nitric oxide (NO)/cGMP postsynaptic pathway. These effects are neuroprotective because they limit energy failure. The present findings may be helpful in the preclinical search for therapeutic strategies aimed at preventing acute and long-term neurological consequences of postnatal asphyxia.

## 1. Introduction

Perinatal asphyxia (or hypoxia–ischemia) is a lack of blood flow and/or gas exchange to the fetus/neonate immediately before, during, or after the birth process, due to compromised placental and/or pulmonary function. The incidence is between one and three per 1000 live births in developed countries, but the rate is up to about ten times higher in developing countries. Fifteen to twenty percent of the affected newborns die (accounting for an estimated 900,000 deaths each year) and up to 25% of survivors remain with permanent neurologic deficits. The significant human and social impact of perinatal asphyxia prompted a large number of experimental and clinical studies, which greatly improved our knowledge in this field. Short-term effects are hypoxic–ischemic encephalopathy and seizures, and long-term consequences include cerebral palsy, epilepsy, and behavioral/cognitive impairments [1,2,3,4]. The risk of cerebral palsy is increased threefold in the presence of neonatal seizures [5]; dyskinetic tetraplegic cerebral palsy is the most common subtype [6].

Notably, only a few studies have focused on asphyxia occurring later in the postnatal period, which can be due to various conditions, including pulmonary, neurological, or cardiovascular abnormalities, or positions of the body that prevent adequate breathing (positional asphyxia). The newborn’s brain continues to develop after birth, and consequently the responsiveness to injuries such as asphyxia progressively changes [7]. The knowledge of the molecular processes involved in the response to asphyxia at defined ages is a necessary condition to offer appropriate targets for therapeutic intervention.

In both perinatal and postnatal asphyxia, the hypoxic insult can cause the immediate (necrosis) and delayed death (apoptosis) of neurons. No established treatment prevents necrosis, but apoptosis can be reduced by therapeutic interventions, which mainly consist of hypothermia to limit secondary energy failure. This treatment improves the outcomes, but its efficacy is limited, and its proper application requires expensive devices that can be prohibitive for some developing countries [8,9]. In an attempt to increase its effectiveness or replace it with new treatments, various promising agents have been used in pre-clinical studies, but the results are not yet satisfactory [10,11,12]. The severity of the long-term consequences is closely related to the amount of neurological damage that occurs within a few minutes of the onset of hypoxia, as a function of the balance between early disruptive and protective hypoxia-induced effects. Therefore, the understanding of the molecular mechanisms underlying the early changes occurring at the age of the hypoxic insult is important for developing appropriate therapeutic strategies.

Accordingly, experimental studies in animal models of hypoxia and hypoxia–ischemia have been performed at different ages [13,14,15,16]. The most used animals were rats and mice, with a minority of researchers studying larger animals such as pigs, sheep, or primates. Most of the studies in rat models have been performed in postnatal days (P) 0–10, and fewer studies after P11, based on comparative analyses suggesting that brain development of neonatal and infant rats up to about P10–13 days corresponds to the third trimester of human gestation. An analysis of experimental data from the literature estimated that the cerebral cortex of a P12–13 rat is developmentally comparable to that of a full-term newborn human [17]. A more recent study based on electrocortical brain activity recordings suggested that P1, P7, and P10 rats correspond to human 23, 30–32, and 40–42 weeks of gestation, respectively [18].

The most widely used animal model of hypoxia–ischemia is the unilateral carotid artery ligation of Rice–Vannucci [19]. Oxygen deprivation in neonatal and infant rats is the most used hypoxia-only model to focus on postnatal asphyxia and asphyxia-induced seizures, and to investigate electrophysiological and molecular mechanisms underlying their acute and long-term consequences [16]. Interestingly, Jensen et al. [20] found that oxygen deprivation induced epileptiform tonic/clonic behavior associated with epileptiform EEG activity significantly more frequently in P10–12 (64%) and P15–17 (62%) than in P5–7 (19%) rats. No epileptiform tonic/clonic behavior was observed in P25–27 and in P50–60 rats. In vitro hypoxia-only models are also extensively used; brain slice preparations proved to be particularly useful, because they allow for the combination of cellular and system level approaches.

Neurons mostly rely on oxidative metabolism for the maintenance of ion homeostasis and membrane potential; accordingly, electrophysiological recordings in brain slices have shown that neuronal membrane potential is highly sensitive to reduced oxygen availability. Depolarization and/or hyperpolarization may occur, depending on the brain region and the type of neuron, and even on the resting potential of the neuron [21]. Hypoxia-induced hyperpolarization is neuroprotective, as being associated with synaptic depression, it decreases energy consumption [22]. Notably, hypoxia-induced synaptic depression appeared later and was less pronounced in CA1 neurons in hippocampal slices from P1–P11 rats than in those from P14–21 and adult rats [23], and in II/III-layer neurons of somatosensory cortex slices from P5–8 and P14–18 rats than in those from adult rats [24,25].

Extracellular adenosine increases in the brain during hypoxia. Most of the studies performed in adult animal preparations show that the resulting increased activation of A_1_ receptors may play a neuroprotective role, mainly by reducing glutamate release and NMDA receptor activation, and by inducing hyperpolarization through inwardly rectifying K^+^ channel activation [26,27,28,29,30,31,32]. The protective effect is more pronounced in the early phase of the hypoxic event, and then it becomes progressively less efficient due to the internalization of A_1_ receptors and the desensitization of A_1_ receptor-mediated responses [33]. Notably, data obtained in infant animals suggest that increased adenosine A_1_ receptor activation may exert adverse effects on the developing brain, rather than neuroprotection [34]. For example, A_1_ receptor stimulation could not prevent ischemic brain damage in 7-day-old rats, likely reflecting the lower number of receptors (23% of the adult levels) [35]. Moreover, a study performed in P3–14 mice exposed to hypoxia showed that these receptors play a prominent role in the development of hypoxia-induced ventriculomegaly [36].

Another interesting player in hypoxia is nitric oxide (NO), which has emerged as both a major neurotoxic agent and a potential therapeutic intervention. Despite the extensive therapeutic use of inhaled NO for pulmonary artery hypertension, its actions during brain hypoxia and ischemia (which coexist with pulmonary artery hypertension in 20–30% of affected infants) are still unclear, due to the high variability of the effects produced [37]. The effects may differ according to various factors, including age, temporal stage after hypoxia onset, brain region and cell type by which it is produced, NO synthase (NOS) isoform involved, amount of NO produced, cellular redox state, and underlying disease processes [37,38,39,40]. Simplifying a much more complex scenario, data obtained so far suggest that NO produced by endothelial NOS mainly plays a neuroprotective role, by maintaining cerebral blood flow (vasodilatory action) and by inhibiting platelet and leukocyte adhesion, whereas NO produced by inducible and neuronal NOS tends to be neurotoxic, leading to cascade reactions of excitotoxicity, inflammation, and apoptosis. Consequently, clinical trials with selective neuronal and inducible NOS inhibitors as potential agents for the treatment of hypoxic encephalopathy are now ongoing [39]. Notably, NO is a key signaling molecule that plays a critical role in a wide range of physiological functions in the brain, including synaptic plasticity and memory formation [41,42,43]. In hippocampal slices from adult rats, NO was found to contribute to synaptic depression induced by prolonged (8–45 min) but not by brief (2–3 min) hypoxic events in CA1 neurons [44]. In the same preparation, synaptic depression induced by a selective agonist of adenosine A_1_ receptors resulted to be partly mediated by the NO/cGMP pathway [45].

Surprisingly, electrophysiological changes induced by hypoxia in the infant motor cortex have not been investigated so far, despite the critical involvement of this cortical area in epilepsy. In the present study, to shed light on the acute effects of the decrease in oxygen availability in this specific neuronal network, primary motor cortex slices obtained from the brains of infant rats were exposed to 10 min of hypoxia. We choose the age window P16–18 as a model of human postnatal asphyxia because it is characterized by a high incidence of hypoxia-induced seizures associated with epileptiform motor behavior [20]. Extracellular field potentials evoked by horizontal pathway stimulation were recorded in layers II/III of the primary motor cortex before, during, and after the hypoxic event to study the effect on glutamatergic synaptic transmission and neuronal excitability.

## 2. Materials and Methods

### 2.1. Animals 

Twenty-nine (*n* = 29) P16–18 infant male Sprague Dawley CD IGS rats (Charles River Laboratoires Italia, Calco, Italy) were used in this study. Animals were treated in accordance with the European Community Directives 86/609/EEC and 2010/63/EU, and the 3R concept has been considered when planning the experiments. The animal study protocol was approved by the Ethical Committee of the University of Bologna. Rats were individually housed under controlled conditions (temperature: 24 ± 1 °C; humidity: 50 ± 5%), maintained on a 12:12 h light-dark cycle, and fed ad libitum.

### 2.2. Slice Preparation 

The experiments were carried out in coronal brain slices including the primary motor cortex (M1). The slices were prepared as previously described [46]. Briefly, rats were deeply anaesthetized using halothane and rapidly decapitated. Their brains were rapidly removed and immersed in ice-cold low-sodium, high-sucrose solution containing (in mM): 212.7 sucrose, 26.0 NaHCO_3_, 2.6 KCl, 1.23 NaH_2_PO_4_, 2.0 MgSO_4_, 10 dextrose, and 2.0 CaCl_2_, bubbled with a mixture of 95% O_2_ and 5% CO_2_ at pH 7.4. Coronal slices, 400 µm thick, were cut using an oscillating tissue slicer (FHC, Bowdoin, ME, USA), beginning ~2 mm caudal to the frontal pole. Slices were collected in a range from 3.7 to 1.7 mm anterior to Bregma [47] in artificial cerebro-spinal fluid (ACSF) of the following composition (in mM): 126.0 NaCl, 26.0 NaHCO3, 3.0 KCl, 1.25 NaH_2_PO_4_, 1.0 MgSO_4_, 2.0 CaCl_2_, and 10.0 dextrose, bubbled with a mixture of 95% O_2_ and 5% CO_2_ at pH 7.4 and maintained at room temperature. All chemicals were purchased from Sigma Adrich - Merck KGaA (Darmstadt, Germany). After a recovery time of at least 1 h, the slices were transferred into a submersion recording chamber perfused (3 mL/min) with warm (34 °C) ACSF and left undisturbed for at least another hour before starting recording.

### 2.3. Extracellular Field Potential Recording

Field excitatory postsynaptic potentials (fEPSPs) evoked by glutamatergic afferent pathway stimulation were recorded using glass micropipettes filled with 2.0 M NaCl (1–3 MΩ) and connected to a DC current amplifier by an Ag/AgCl electrode. Recording micropipettes were positioned in layers II/III of the primary motor cortex, 200–400 µm below the cortical surface and 2.5–4.5 mm lateral to the midline. Horizontal cortical pathways were stimulated with a concentric bipolar electrode (70–80 KΩ; FHC, Bowdoin, ME, USA) located in cortical layers II/III, at ~500 µm from the recording electrode [48]. Constant-current square pulses (0.2 ms) were applied at 0.1 Hz using the stimulus generator Master 8 (AMPI, Jerusalem, Israel); stimulus intensity was adjusted to the value needed to elicit the maximal synaptic response (we chose this intensity to be able to also induce a non-synaptic response large enough to be measured accurately). Only slices in which we could elicit a synaptic response of at least 0.8 mV amplitude were considered in this study. Before hypoxia induction, the perfusion was switched to an ACSF containing the GABA_A_ receptor inhibitor (-)-bicuculline methobromide (BMI, 2 µM) and, when indicated, 1,3-dipropyl-8-cyclopentylxanthine (DPCPX, 50 nM), 1H-[1,2,4]oxadiazolo [4,3-a]quinoxalin-1-one (ODQ, 100 µM), and Nω-nitro-l arginine methyl ester (L-NAME, 2 mM) or DPCPX (50 nM) plus L-NAME (2 mM). In order to identify non-synaptic and synaptic components of the fEPSPs, at the end of all the experiments the perfusion was switched to an ACSF containing the AMPA and kainate glutamate receptors blocker 6-cyano-7-nitroquinoxaline-2,3-dione (CNQX, 20 µM).

### 2.4. Hypoxia Induction

Hypoxia was induced when the amplitude of the synaptic response was stable (none of the values exceeding the average by more than 10%): slices were perfused with ACSF equilibrated with 95% N_2_ and 5% CO_2_ (instead of 95% O_2_ and 5% CO_2_). Changes in O_2_ pressure (pO_2_) were monitored with an O_2_ electrode (WPI, Sarasota, FL, USA) placed in the perfusing ACSF within the recording chamber. In order to avoid the onset of anoxic depolarization (AD) we set an anoxic perfusion time of 10 min (AD events were never observed in these conditions).

### 2.5. Agents and Chemicals

BMI, CNQX, DPCPX, ODQ, and TTX were purchased from Tocris - Biotechne (Minneapolis, MN, USA), and L-NAME from Sigma Adrich - Merck KGaA (Darmstadt, Germany). DPCPX was made in a 5 mM stock solution of 99% dimethyl sulfoxide (DMSO) and 1% 1 M NaOH; ODQ was dissolved in 99% DMSO. DPCPX and ODQ solutions were then diluted in ACSF. TTX was dissolved in distilled water and then diluted in ACSF. All the other drugs were directly dissolved in ACSF.

### 2.6. Data Analysis and Statistics

*Responses to single-pulse stimulation.* We measured the amplitude of the non-synaptic and the synaptic components of the fEPSPs by using the software Axon Clampfit 7.0 (Molecular Devices, San Jose, CA, USA). Six consecutive responses were pooled in each minute data and expressed as mean ± standard deviation. For each slice, data were normalized taking as 100% the averaged of the values obtained during the last ten minutes before hypoxia onset. Statistical analysis within and among groups was performed by the one-way analysis of variance (ANOVA) followed by the post hoc Tukey’s multiple comparison test.

*Responses to paired-pulse stimulation*. We measured the amplitude of the synaptic component of the fEPSPs evoked by two pulses delivered at a 50 ms interval. The average of six consecutive responses to the second pulse (R2) was divided by the average of six consecutive responses to the first one (R1) and expressed as paired-pulse ratio (mean ± standard deviation). Statistical analysis between pre-hypoxia and hypoxia values was performed using Student’s *t*-test.

## 3. Results

Pyramidal cells in layer II/III make mono-synaptic connections via long horizontal collaterals with the proximal dendrites of layer II/III cells belonging to distant columns [48]. We found that the stimulation of these axon collaterals generates an fEPSP containing: (a) a first short-latency (1.4 ± 0.4 ms; *n* = 18) negative component, due to neuronal activation as it was sensitive to TTX (1 µM), but non-synaptic being resistant to CNQX (20 µM); (b) a second negative-going wave (latency: 2.8 ± 1.0 ms; *n* = 23), which is the glutamatergic synaptic component as it was sensitive to CNQX (20 µM).

### 3.1. Hypoxia-Induced Depression of Neuronal Excitability and Glutamatergic Synaptic Transmission

**Figure 1:**
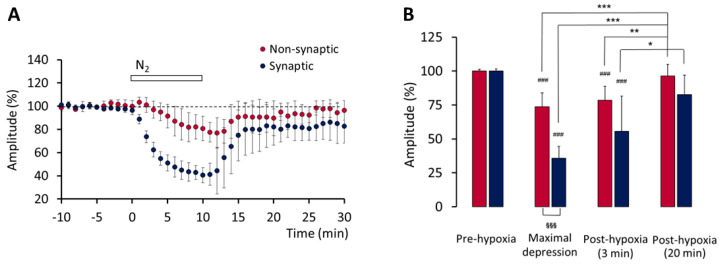
Hypoxia-induced changes on fEPSPs recorded in layers II/III of the primary motor cortex, in brain slices from infant rats. (**A**) Time course of the effect of hypoxia on the amplitude of non-synaptic (red symbols) and synaptic (blue symbols) components of the fEPSPs (*n* = 5). (**B**) Amplitude of non-synaptic (red histograms) and synaptic (blue histograms) components of the fEPSPs: comparison of the mean values (± SD) recorded during 10 min before hypoxia onset (pre-hypoxia), during the minute corresponding to the maximal hypoxia-induced depressive effect, and during min 3 and min 20 after re-oxygenation onset. ### *p* < 0.001, *** *p* < 0.001, ** *p* < 0.01, * *p* < 0.05; §§§ *p* < 0.001 (vs. the columns indicated).

The 10 min perfusion with anoxic ACSF caused a gradual fall in O_2_ tension (pO_2_) within the recording chamber, and both the depressed non-synaptic and synaptic components of the fEPSPs; pO_2_ gradually decreased to about 20% of the control values, then rapidly recovered. The non-synaptic component of the fEPSPs was gradually depressed during and after hypoxia and reached the minimal mean value about 12 min after hypoxia onset. The synaptic component was more strongly depressed and reached the minimal mean value about 10 min after hypoxia onset. The effect on the two components of the fEPSPs is shown in Figure 1. The statistical analysis revealed that the means of the minimal values were significantly different with respect to the means of pre-hypoxia baseline values (*p* < 0.001), and that the mean of non-synaptic minimal values was significantly different with respect to the mean of the synaptic minimal values (*p* < 0.001) (Figure 1B). Both fEPSPs components recovered after 20 min of re-oxygenation (Figure 1B).

### 3.2. Involvement of Adenosine A_1_ Receptors and the NO/cGMP Pathway in the Depressive Effects of Hypoxia

To evaluate whether the adenosine A_1_ receptor- and the NO/cGMP pathways were involved in the depressive effects of hypoxia, we compared the fEPSP components recorded in control conditions with those recorded in slices pre-treated with the adenosine A_1_ receptor antagonist DPCPX (50 nM), the NO synthase inhibitor L-NAME (2 mM), or the NO-sensitive soluble guanylyl cyclase (GC) inhibitor ODQ (100 µM).

**Figure 2:**
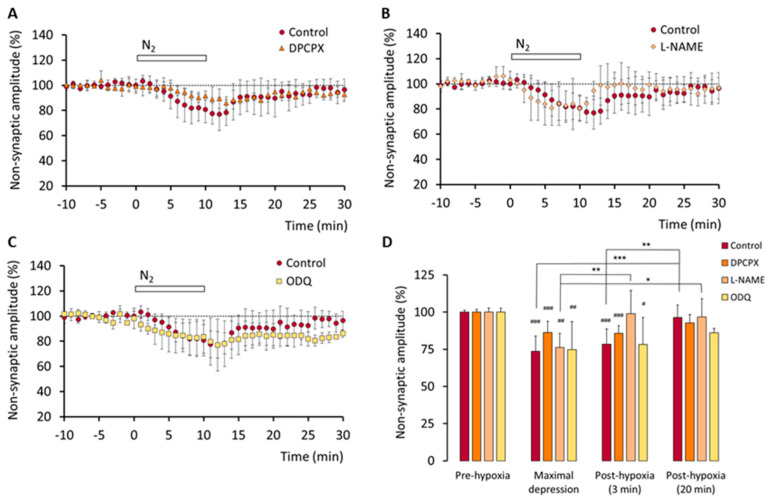
Adenosine A_1_ receptors and NO/cGMP are not involved in hypoxia-induced depression of the non-synaptic component of the fEPSPs. Time course of hypoxia effect on the amplitude of the non-synaptic peak in control conditions (*n* = 5) and after pre-treatment with: (**A**) the A_1_ receptors antagonist DPCPX (50 nM; *n* = 3); (**B**) the NO synthase inhibitor L-NAME (2 mM; *n* = 5); (**C**) the NO-sensitive GC inhibitor ODQ (100 µM; *n* = 4). (**D**) Comparison of the mean amplitude (± SD) of the non-synaptic peak recorded during the 10 min before hypoxia onset (pre-hypoxia), during the minute corresponding to the maximal hypoxia-induced depressive effect, and during min 3 and min 20 after re-oxygenation onset in the different conditions indicated. ### *p* < 0.001, ## *p* < 0.01, # *p* < 0.05 (vs. pre-hypoxia). *** *p* < 0.001, ** *p* < 0.01, * *p* < 0.05 (vs. the columns indicated).

As shown in Figure 2, the effect of hypoxia on the amplitude of the non-synaptic component of the fEPSP (which is considered an index of neuronal excitability) was not significantly different in the control slices (*n* = 5) with respect to those pre-treated with DPCPX (*n* = 4; Figure 2A), L-NAME (*n* = 5; Figure 2B), or ODQ (*n* = 4; Figure 2C). These results suggest that the A_1_ receptors and the NO/cGMP pathway do not play a major role in the depressive effect of hypoxia on neuronal excitability. Comparisons among the means of the values recorded during pre-hypoxia, maximal depression, and after 3 and 20 min of re-oxygenation, in control conditions and in the presence of DPCPX, L-NAME or ODQ, are shown in Figure 2D.

**Figure 3:**
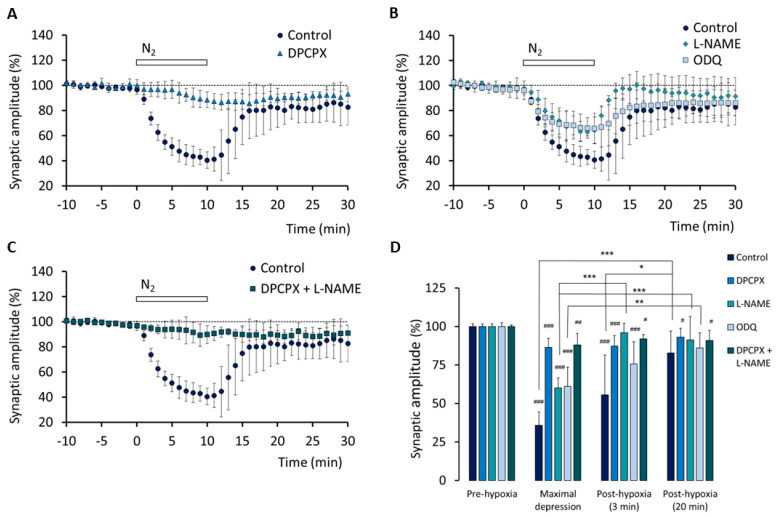
Involvement of adenosine A_1_ receptors and NO/cGMP in hypoxia-induced depression of the synaptic component of the fEPSPs. Time course of hypoxia effect on the amplitude of the synaptic peak in control conditions (*n* = 5) and after pre-treatment with: (**A**) the A_1_ receptors antagonist DPCPX (50 nM; *n* = 4); (**B**) the NO synthase inhibitor L-NAME (2 mM; *n* = 5) or the NO-sensitive GC inhibitor ODQ (100 µM; *n* = 5); (**C**) both DPCPX (50 nM) and L-NAME (2 mM) (*n* = 4). (**D**) Comparison of the mean amplitude (± SD) of the synaptic peak recorded during the 10 min before hypoxia onset (pre-hypoxia), during the minute corresponding to the maximal hypoxia-induced depressive effect, and during min 3 and min 20 after re-oxygenation onset in the different conditions indicated. ### *p* < 0.001, ## *p* < 0.01; # *p* < 0.05 (vs. pre-hypoxia). *** *p* < 0.001, ** *p* < 0.01, * *p* < 0.05 (vs. the columns indicated).

By contrast, the peak amplitude of the synaptic component of the fEPSPs was much less depressed by hypoxia in slices pre-treated with DPCPX (*n* = 4) than in control slices: the means of the values were significantly different at the maximal depression (*p* < 0.001) and after 3 min of re-oxygenation (*p* < 0.05) (Figure 3A,D). The effect of DPCPX suggests a major role of adenosine acting on A_1_ receptors in the depressive effect of hypoxia on glutamatergic transmission. Comparisons among the means of the values recorded during pre-hypoxia, maximal depression, and after 3 and 20 min of re-oxygenation, in control conditions and in the presence of DPCPX (or the other drugs), are shown in Figure 3D.

Figure 3B shows that also in the slices pre-treated with L-NAME (*n* = 5), the synaptic peak amplitude was less depressed by hypoxia than in the control slices: the means of the values were significantly different at the maximal depression (*p* < 0.01) and after 3 min of re-oxygenation (*p* < 0.01) (Figure 3B,D). The effect of L-NAME suggests an involvement of NO in the depressive effect of hypoxia on glutamatergic transmission. The comparison between the effects of DPCPX and L-NAME (cf. Figure 3A,B and see Figure 3D) suggests that the NO contribution to hypoxia-induced synaptic depression is smaller than that of adenosine acting through A_1_ receptors.

In addition, Figure 3B shows that also in ODQ-treated slices (*n* = 5), the synaptic peak amplitude was less depressed by hypoxia than in the control slices; the means of the values were significantly different at the maximal depression (*p* < 0.01), but not after 3 min of re-oxygenation (Figure 3B,D). Nevertheless, the comparison between the effects of L-NAME and ODQ (Figure 3B) suggests a major involvement of the NO-sensitive GC in the effect of NO on synaptic transmission.

In a series of experiments, the slices (*n* = 4) were treated with both DPCPX (50 nM) and L-NAME (2 mM). We found that the synaptic fEPSP component was depressed by hypoxia as in DPCPX-treated slices (cf. Figure 3A,C,D): the means of the values were significantly different vs. those recorded in control slices at maximal depression (*p* < 0.001) and after 3 min of re-oxygenation (*p* < 0.05).

### 3.3. Involvement of Changes at Pre-synaptic Level in Hypoxia-Induced Depression of Synaptic Transmission

To assess whether hypoxia-induced depression of synaptic transmission involved changes at the pre-synaptic level, we used the paired-pulse paradigm, which provides a reliable indication of pre-synaptic events [49,50].

**Figure 4:**
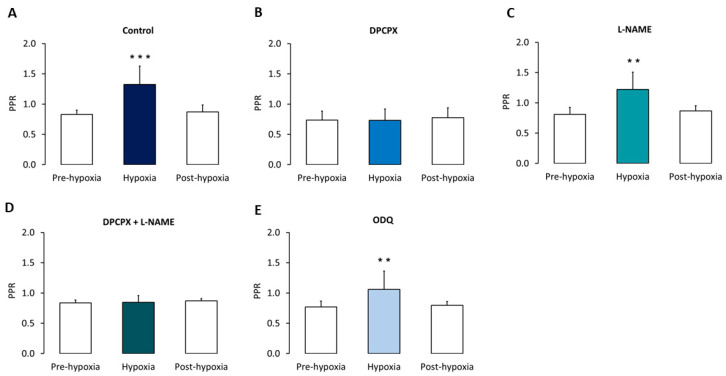
Paired-pulse ratio changes induced by hypoxia. (**A**–**E**) Comparison of the mean (± SD) paired-pulse ratio (PPR) of synaptic peak amplitudes (average of six consecutive responses to the second pulse (R2) divided by the average of six consecutive responses to the first one (R1)) recorded during the last minute before hypoxia onset (pre-hypoxia), during the minute corresponding to the maximal hypoxia-induced depressive effect of the response evoked by the first pulse (hypoxia), and during min 20 after re-oxygenation onset (post-hypoxia), in: (**A**) control conditions (*n* = 5); (**B**) after pre-treatment with the A_1_ receptors antagonist DPCPX (50 nM; *n* = 4); (**C**) after pre-treatment with the NO synthase inhibitor L-NAME (2 mM; *n* = 5); (**D**) after pre-treatment with both DPCPX (50 nM) and L-NAME (2 mM) (*n* = 4); (**E**), after pre-treatment with the NO-sensitive GC inhibitor ODQ (100 µM; *n* = 5). *** *p* < 0.005, ** *p* < 0.01.

Under normoxic conditions (*n* = 5), using a 50 ms inter-stimulus interval, the paired-pulse ratio (PPR) of synaptic amplitudes (responses to the second pulse divided by responses to the first one) was 0.831 ± 0.069 (i.e., paired-pulse depression). During hypoxia, PPR significantly (*p* < 0.005) increased (1.325 ± 0.304; i.e., paired-pulse facilitation), suggesting a decrease in glutamate release. PPR returned to pre-hypoxia values after re-oxygenation (0.871 ± 0.114) (Figure 4A).

In DPCPX-treated slices (*n* = 4), PPR did not increase during hypoxia, suggesting a major involvement of adenosine, which through pre-synaptic A_1_ receptors decreased glutamate release (Figure 4B).

By contrast, in L-NAME- (*n* = 5) and ODQ- (*n* = 5) treated slices, PPR significantly (*p* < 0.01 and *p* < 0.05, respectively) increased during hypoxia, suggesting that NO does not play a significant role in the hypoxia-induced decrease in glutamate release. PPR recovered after re-oxygenation (Figure 4C,E).

In DPCPX + L-NAME (*n* = 4) -treated slices, PPR did not increase during hypoxia (Figure 4D), as observed in slices only treated with DPCPX.

## 4. Discussion

In order to investigate the effects induced in the motor cortex by an asphyxia event occurring in the postnatal period, we exposed brain slices from infant rats in the age window P16–18, which is characterized by high incidence of hypoxia-induced seizures associated with epileptiform motor behavior, to 10 min of hypoxia [20].

The results obtained show that the hypoxic event caused a reversible depression of neuronal excitability and glutamatergic synaptic transmission (maximal depression: 26 and 64%, respectively) in the primary motor cortex. The effect of hypoxia on synaptic transmission is consistent with previous data obtained in CA1 neurons in rat hippocampal slices exposed to 2–4 min of hypoxia; compared to our results, the maximal synaptic depression was lesser in hippocampal slices from P1 and P4 rats (42 and 52%, respectively), and greater in those from P11, P14, P21, and adult rats (72, 100, 98, and 89%, respectively) [23]. The synaptic depression observed in the present study is also consistent with previous results obtained in II/III-layer neurons in rat somatosensory cortex slices exposed to 2.5 [24] or 20 [25] min of hypoxia; compared to our results, the maximal synaptic depression was lesser in both studies, at all the ages considered: P5–8 (10 and 3%, respectively), P14–18 (15 and 42%, respectively), and adulthood (42% and 55%, respectively). Since about 33–50% of cerebral oxygen is used for synaptic transmission [22], its depression is strongly neuroprotective: by decreasing the mismatch between energy requirement and supplies, it contributes to the prevention of the early and long-term neurological consequences of hypoxia. Present results and the previous ones obtained in the hippocampus [23] and in the somatosensory cortex [24,25] suggest that this protective system develops and progressively becomes more and more effective, but at different speeds and reaching a different maximum level of efficacy in different brain regions in adulthood; the hippocampus appears to be the most protected brain region.

The strong effect of hypoxia on synaptic responses to paired-pulse stimulation (from depression to facilitation) observed in the present experiments suggests that synaptic depression is at least partly due to the inhibition of glutamate release. This view is further supported by the following evidence, which suggest the involvement of adenosine acting on pre-synaptic A_1_ receptors: the specific A_1_ antagonist DPCPX prevented the hypoxia-induced inhibition of synaptic responses, as well as the effect of hypoxia on synaptic responses to paired-pulse stimulation. Moreover, a previous observation in hippocampal slices from adult mice provided direct evidence that the neuroprotective role of adenosine during hypoxia depend on inhibition of synaptic transmission by the activation of presynaptic A_1_ receptors: the focal deletion of these receptors on the Schaffer collateral input slowed the depression of CA3 fEPSPs in response to hypoxia and impaired the recovery of the fEPSPs after hypoxia [29]. In our experiments, we cannot exclude that post-synaptic adenosine A_1_ receptors activation might contribute to synaptic inhibition, through the inhibition of NMDA receptors and/or activation of inwardly rectifying K^+^ channels. However, the latter contribution appears negligible in present experimental conditions, as the specific A_1_ antagonist DPCPX did not prevent a hypoxia-induced decrease in neuronal excitability. Regardless of the site of action, present results provide the first evidence of neuroprotection by adenosine, via A_1_ receptors, against energy failure induced by hypoxia in the primary motor cortex of infant rats. This evidence appears particularly relevant, as previous studies had shown that the adenosine activation of A_1_ receptors during hypoxia induced neurotoxic rather than neuroprotective effects in the developing brain [34,35,36].

The results obtained in the presence of the NO synthase inhibitor L-NAME suggest that NO was also involved in the hypoxia-induced depression of synaptic transmission, in accordance with previous observations in hippocampal slices from adult rats [44,51]. In our experiments, the NO contribution to synaptic depression was smaller than that of adenosine acting on A_1_ receptors. Physiological NO signal transduction mainly occurs through the activation of intracellular GC-coupled receptors, leading to cGMP formation [52,53]. Present data suggest the involvement of the cGMP pathway in the contribution of NO to the depressive effect on synaptic transmission, since it is completely prevented by the GC inhibitor ODQ. This is consistent with the previous observation that an NO donor depresses synaptic transmission in hippocampal CA1 through an increase in cGMP [54], and with a recent study partly performed in our laboratory showing that the NO/cGMP pathway in the rat perirhinal cortex is involved in a form of long-term depression of glutamatergic synaptic transmission that is crucial for visual recognition memory [42]. Here, we also found that the blockade of either NO or cGMP synthesis does not prevent hypoxia-induced changes of synaptic responses to paired-pulse stimulation (from depression to facilitation), suggesting that NO depressed synaptic transmission by acting at a post-synaptic level rather than on glutamate release, in agreement with previous findings [54,55]. In hippocampal slices from adult rats, synaptic depression induced by a selective agonist of adenosine A_1_ receptors resulted to be partly mediated by the NO/cGMP pathway [45]. The present results suggest that the two depressive mechanisms are instead independent in the motor cortex, because they act in different synaptic sites. Apart from the mechanism of action, the present data provide the first evidence in support of the hypothesis that in addition to adenosine, NO may also contribute to limiting the energy failure induced by hypoxia in the primary motor cortex of infant rats. This potential neuroprotective action should be considered among the many contrasting effects induced by NO during and after hypoxic events.

The present results show that hypoxia also induced a minor and reversible A_1_ receptor- and NO-independent depression of neuronal excitability; this effect contributes to neuroprotection by reducing action potential firing. In accordance with our data, previous studies in somatosensory cortex slices from adult rats have shown that hypoxia induces hyperpolarization and a decrease in input resistance, possibly through the activation of ATP-sensitive K^+^ conductance [56,57]. Studies in hippocampal slices from adult rats have shown that the CA1 antidromic population spike amplitude is not (or is only slightly) depressed by hypoxia [58,59,60]; membrane hyperpolarization and decrease in input resistance were also observed [61,62].

## 5. Conclusions

The results of the present study provide the first evidence that hypoxia causes a reversible depression of glutamatergic synaptic transmission and neuronal excitability in primary motor cortex slices from infant rats. Data obtained in the presence of specific blockers suggest that synaptic depression was mediated by adenosine acting on presynaptic A_1_ receptors to decrease glutamate release, and by an NO/cGMP postsynaptic pathway. Depression of synaptic transmission and neuronal excitability is neuroprotective because it limits energy failure: by decreasing the mismatch between energy needs and supplies, it contributes to the prevention of early and long-term neurological consequences of hypoxia. The present findings might be helpful in the search for therapeutic strategies aimed at preventing acute and long-term neurological consequences of postnatal asphyxia.

## Data Availability

The data presented in this study are available on request from the corresponding authors.

## References

[1] Kurinczuk J.J., White-Koning M., Badawi N. (2010). Epidemiology of neonatal encephalopathy and hypoxic-ischaemic encephalopathy. Early Hum. Dev..

[2] Ahearne C.E., Boylan G.B., Murray D.M. (2016). Short and long term prognosis in perinatal asphyxia: An update. World J. Clin. Pediatr..

[3] Rocha-Ferreira E., Hristova M. (2016). Plasticity in the neonatal brain following hypoxic-ischaemic injury. Neural Plast..

[4] Odd D., Heep A., Luyt K., Draycott T. (2017). Hypoxic-ischemic brain injury: Planned delivery before intrapartum events. J. Neonatal Perinatal Med..

[5] Dixon G., Badawi N., Kurinczuk J.J., Keogh J.M., Silburn S.R., Zubrick S.R., Stanley F.J. (2002). Early developmental outcomes after newborn encephalopathy. Pediatrics.

[6] Rennie J.M., Hagmann C.F., Robertson N.J. (2007). Outcome after intrapartum hypoxic ischaemia at term. Semin. Fetal Neonatal Med..

[7] Jensen F.E. (2006). Developmental factors regulating susceptibility to perinatal brain injury and seizures. Curr. Opin. Pediatr..

[8] Jacobs S.E., Berg M., Hunt R., Tarnow-Mordi W.O., Inder T.E., Davis P.G. (2013). Cooling for newborns with hypoxic ischaemic encephalopathy. Cochrane Database Syst. Rev..

[9] Rivero-Arias O., Eddama O., Azzopardi D., Edwards A.D., Strohm B., Campbell H. (2019). Hypothermia for perinatal asphyxia: Trial-based resource use and costs at 6–7 years. Arch. Dis. Child. Fetal Neonatal.

[10] Varani K., Vincenzi F., Merighi S., Gessi S., Borea P.A. (2017). Biochemical and pharmacological role of A_1_ adenosine receptors and their modulation as novel therapeutic strategy. Adv. Exp. Med. Biol..

[11] Solevåg A.L., Schmölzer G.M., Cheung P.Y. (2019). Novel interventions to reduce oxidative-stress related brain injury in neonatal asphyxia. Free Radic. Biol. Med..

[12] Tetorou K., Sisa C., Iqbal A., Dhillon K., Hristova M. (2021). Current therapies for neonatal hypoxic-ischaemic and infection-sensitised hypoxic-ischaemic brain damage. Front. Synaptic Neurosci..

[13] Nieber K. (1999). Hypoxia and neuronal function under in vitro conditions. Pharmacol. Ther..

[14] Krnjević K. (1999). Early effects of hypoxia on brain cell function. Croat. Med. J..

[15] Peña F., Ramirez J.M. (2005). Hypoxia-induced changes in neuronal network properties. Mol. Neurobiol..

[16] Millar L.J., Shi L., Hoerder-Suabedissen A., Molnár Z. (2017). Neonatal hypoxia ischaemia: Mechanisms, models, and therapeutic challenges. Front. Cell. Neurosci..

[17] Romijn H.J., Hofman M.A., Gramsbergen A. (1991). At what age is the developing cerebral cortex of the rat comparable to that of the full-term newborn human baby?. Early Hum. Dev..

[18] Tucker A.M., Aquilina K., Chakkarapani E., Hobbs C.E., Thoresen M. (2009). Development of amplitude-integrated electroencephalography and interburst interval in the rat. Pediatr. Res..

[19] Rice J.E., Vannucci R.C., Brierley J.B. (1981). The influence of immaturity on hypoxic-ischemic brain damage in the rat. Ann. Neurol..

[20] Jensen F.E., Applegate C.D., Holtzman D., Belin T.R., Burchfiel J.L. (1991). Epileptogenic effect of hypoxia in the immature rodent brain. Ann. Neurol..

[21] Englund M., Hyllienmark L., Brismar T. (2001). Chemical hypoxia in hippocampal pyramidal cells affects membrane potential differentially depending on resting potential. Neuroscience.

[22] Astrup J. (1982). Energy-requiring cell functions in the ischemic brain: Their critical supply and possible inhibition in protective therapy. J. Neurosurg..

[23] Cherubini E., Ben-Ari Y., Krnjević K. (1989). Anoxia produces smaller changes in synaptic transmission, membrane potential, and input resistance in immature rat hippocampus. J. Neurophysiol..

[24] Luhmann H.J., Kral T., Heinemann U. (1993). Influence of hypoxia on excitation and GABAergic inhibition in mature and developing rat neocortex. Exp. Brain Res..

[25] Luhmann H.J., Kral T. (1997). Hypoxia-induced dysfunction in developing rat neocortex. J. Neurophysiol..

[26] Canhão P., de Mendonça A., Ribeiro J.A. (1994). 1,3-Dipropyl-8-cyclopentylxanthine attenuates the NMDA response to hypoxia in the rat hippocampus. Brain Res..

[27] De Mendonça A., Sebastião A.M., Ribeiro J.A. (1995). Inhibition of NMDA receptor-mediated currents in isolated rat hippocampal neurones by adenosine A_1_ receptor activation. Neuroreport.

[28] De Mendonça A., Sebastião A.M., Ribeiro J.A. (2000). Adenosine: Does it have a neuroprotective role after all?. Brain Res. Rev..

[29] Arrigoni E., Crocker A.J., Saper C.B., Greene R.W., Scammell T.E. (2005). Deletion of presynaptic adenosine A_1_ receptors impairs the recovery of synaptic transmission after hypoxia. Neuroscience.

[30] Corcoran A., O’Connor J.J. (2013). Hypoxia-inducible factor signalling mechanisms in the central nervous system. Acta Physiol..

[31] Kawamura M., Ruskin D.N., Masino S.A. (2019). Adenosine A_1_ receptor-mediated protection of mouse hippocampal synaptic transmission against oxygen and/or glucose deprivation: A comparative study. J. Neurophysiol..

[32] Zhang Y., Cao H., Qiu X., Xu D., Chen Y., Barnes G.N., Tu Y., Gyabaah A.T., Gharbal A., Peng C. (2020). Neuroprotective effects of adenosine A1 receptor signaling on cognitive impairment induced by chronic intermittent hypoxia in mice. Front. Cell. Neurosci..

[33] Coelho J.E., Rebola N., Fragata I., Ribeiro J.A., de Mendonça A., Cunha R.A. (2006). Hypoxia-induced desensitization and internalization of adenosine A_1_ receptors in the rat hippocampus. Neuroscience.

[34] Kashfi S., Ghaedi K., Baharvand H., Nasr-Esfahani M.H., Javan M. (2017). A_1_ adenosine receptor activation modulates central nervous system development and repair. Mol. Neurobiol..

[35] Adén U., Leverin A.L., Hagberg H., Fredholm B.B. (2001). Adenosine A_1_ receptor agonism in the immature rat brain and heart. Eur. J. Pharmacol..

[36] Turner C.P., Seli M., Ment L., Stewart W., Yan H., Johansson B., Fredholm B.B., Blackburn M., Rivkees S.A. (2003). A_1_ adenosine receptors mediate hypoxia-induced ventriculomegaly. Proc. Natl. Acad. Sci. USA.

[37] Angelis D., Savani R., Chalak L. (2021). Nitric oxide and the brain. Part 1: Mechanisms of regulation, transport and effects on the developing brain. Pediatr. Res..

[38] Liu H., Li J., Zhao F., Wang H., Qu Y., Mu D. (2015). Nitric oxide synthase in hypoxic or ischemic brain injury. Rev. Neurosci..

[39] Albrecht M., Zitta K., Groenendaal F., van Bel F., Peeters-Scholte C. (2019). Neuroprotective strategies following perinatal hypoxia-ischemia: Taking aim at NOS. Free Radic. Biol. Med..

[40] Angelis D., Savani R., Chalak L. (2021). Nitric oxide and the brain. Part 2: Effects following neonatal brain injury—Friend or foe?. Pediatr. Res..

[41] Schuman E.M., Madison D.V. (1991). A requirement for the intercellular messenger nitric oxide in long-term potentiation. Science.

[42] Tamagnini F., Barker G., Warburton E.C., Burattini C., Aicardi G., and Bashir Z.I. (2013). Nitric oxide-dependent long-term depression but not endocannabinoid-mediated long-term potentiation is crucial for visual recognition memory. J. Physiol..

[43] Chachlaki K., Prevot V. (2020). Nitric oxide signalling in the brain and its control of bodily functions. Br. J. Pharmacol..

[44] Zhu P.J., Erdemli G. (1995). Nitric oxide may be a mediator of effects of prolonged but not brief anoxia in CA1 neurons in slices. Neuropharmacology.

[45] Pinto I., Serpa A., Sebastião A.M., Cascalheira J.F. (2016). The Role of cGMP on Adenosine A_1_ Receptor-mediated Inhibition of Synaptic Transmission at the Hippocampus. Front. Pharmacol..

[46] Hess G., Aizenman C.D., Donoghue J.P. (1996). Conditions for the induction of long-term potentiation in layer II/III horizontal connections of the rat motor cortex. J. Neurophysiol..

[47] Paxinos G., Watson C. (2007). The Rat Brain in Stereotaxic Coordinates.

[48] Aroniadou V.A., Keller A. (1993). The patterns and synaptic properties of horizontal intracortical connections in the rat motor cortex. J. Neurophysiol..

[49] Hess G., Kuhnt U., Voronin L.L. (1987). Quantal analysis of paired-pulse facilitation in guinea pig hippocampal slices. Neurosci. Lett..

[50] Saviane C., Savtchenko L.P., Raffaelli G., Voronin L.L., Cherubini E. (2002). Frequency-dependent shift from paired-pulse facilitation to paired-pulse depression at unitary CA3-CA3 synapses in the rat hippocampus. J. Physiol..

[51] Wallis R.A., Panizzon K., Wasterlain C.G. (1992). Inhibition of nitric oxide synthase protects against hypoxic neuronal injury. Neuroreport.

[52] Southam E., Garthwaite J. (1993). The nitric oxide-cyclic GMP signalling pathway in rat brain. Neuropharmacology.

[53] Denninger J.W., Marletta M.A. (1999). Guanylate cyclase and the NO/cGMP signaling pathway. Biochim. Biophys. Acta.

[54] Boulton C.L., Irving A.J., Southam E., Potier B., Garthwaite J., Collingridge G.L. (1994). The nitric oxide-cyclic GMP pathway and synaptic depression in rat hippocampal slices. Eur. J. Neurosci..

[55] Garthwaite J. (2005). Dynamics of cellular NO-cGMP signaling. Front. Biosci..

[56] Rosen A.S., Morris M.E. (1991). Depolarizing effects of anoxia on pyramidal cells of rat neocortex. Neurosci. Lett..

[57] Luhmann H.J., Heinemann U. (1992). Hypoxia-induced functional alterations in adult rat neocortex. J. Neurophysiol..

[58] Fowler J.C. (1989). Adenosine antagonists delay hypoxia-induced depression of neuronal activity in hippocampal brain slice. Brain Res..

[59] Doolette D.J., Kerr D.I. (1995). Hyperexcitability in CA1 of the rat hippocampal slice following hypoxia or adenosine. Brain Res..

[60] Sebastião A.M., de Mendonca A., Moreira T., Ribeiro J.A. (2001). Activation of synaptic NMDA receptors by action potential-dependent release of transmitter during hypoxia impairs recovery of synaptic transmission on reoxygenation. J. Neurosci..

[61] Fujimura N., Tanaka E., Yamamoto S., Shigemori M., Higashi H. (1997). Contribution of ATP-sensitive potassium channels to hypoxic hyperpolarization in rat hippocampal CA1 neurons in vitro. J. Neurophysiol..

[62] Erdemli G., Xu Y.Z., Krnjević K. (1998). Potassium conductance causing hyperpolarization of CA1 hippocampal neurons during hypoxia. J. Neurophysiol..

